# Designing an Interdisciplinary Health Course: A Qualitative Study of Undergraduate Students’ Experience of Interdisciplinary Curriculum Design and Learning Experiences

**DOI:** 10.1177/23821205241260488

**Published:** 2024-08-08

**Authors:** Leda Mirbahai, Farhan Noordali, Helen Nolan

**Affiliations:** 1 Warwick Medical School, Gibbet Hill Campus, 2707 University of Warwick, Coventry, UK

**Keywords:** Interdisciplinarity, interdisciplinary learning, integrated assessment, synoptic, case-based learning, holistic learning, student experience, curriculum design

## Abstract

**OBJECTIVES:**

Research into interdisciplinary education, where concrete examples and empirical evidence of interdisciplinary teaching is explored, is limited. Furthermore, there are no standardized guidelines on best practices for designing and implementing an interdisciplinary curriculum. Recently, in healthcare settings there has been a drive to adopt interdisciplinary or transdisciplinary practices, creating a need for training individuals capable of working across discipline-specific boundaries, or to even adopt a transdisciplinary practice. This is partially attributed to recognizing that local and global complex health challenges are interlinked and share common factors and often require a new integrated approach to management. In response, a new interdisciplinary course using a modified snowflake model of interdisciplinary course design was launched at a medical school. The course aimed to provide a broad foundation for lifelong learning with a strong emphasis on the development of knowledge, skills, and professional values essential for interdisciplinary and transdisciplinary practice in applied health promotion for individuals and society.

**METHODS:**

A semi-structured focus group with students (*n* = 15% of the inaugural cohort) having completed at least 1 year of the course was undertaken to investigate student perspectives on best approaches for the development and delivery of interdisciplinary learning and teaching.

**RESULTS:**

Results highlighted the importance of providing training and opportunity for students to practice integration within the curriculum. Additionally, it was noted that including a module to introduce students to different disciplines and guiding students to explore their inherent interconnectedness is essential in helping them develop interdisciplinary thinking and skills. Crucially, the role of integrated assessments was also recognized as fundamental for demonstrating and practicing interdisciplinarity.

**CONCLUSION:**

Overall, this study provides valuable insights and recommendations for educators with the objective of developing interdisciplinary learning in new or existing higher education courses or those seeking to prepare learners for contemporary and emergent societal challenges more generally.

## Introduction

### Outlining interdisciplinary learning

In interdisciplinary education, students are encouraged to analyze the links between different disciplines and synthesize them into a coherent whole to achieve a better understanding of a common theme.^1,2^ However, research into interdisciplinary education, where concrete examples and empirical evidence of interdisciplinary teaching and learning are explored, is limited especially at the undergraduate level.^3–5^ Furthermore, there are no standardized guidelines on best practices for designing and implementing an interdisciplinary curriculum in traditionally monodisciplinary departments and institutes with no or limited infrastructure to support interdisciplinarity. This has led to an array of activities and variation in the implementation of interdisciplinary learning (IDL) experiences.^4,6,7^ Irrespective of this variation in design and implementation of IDL experiences, the perceived benefits associated with IDL have acted as the driving force for traditionally monodisciplinary higher education institutes to promote and design curricular or learning experiences that provide students with the opportunity to cross discipline boundaries and explore the interconnectedness of different disciplines.^8^ It is well recognized that interdisciplinary learning outcomes differ from monodisciplinary learning outcomes. In IDL, students are required to demonstrate “interdisciplinary thinking” which is defined as “the capacity to integrate knowledge and modes of thinking in 2 or more disciplines or established areas of expertise to produce a cognitive advancement, such as explaining a phenomenon, solving a problem, or creating a product, in ways that would have been impossible or unlikely through single disciplinary means.”^9^ Interdisciplinary thinking is partly developed as students experiencing IDL are required to learn in the overlapping space between boundaries of different disciplines which requires complex cognitive skills, heightened ability to recognize bias, tolerance of ambiguity, coping with complexity, criticality, and ability to change perspectives.^5^ However, the knowledge deficit and lack of clear processes for the creation of interdisciplinary education initiatives within traditional monodisciplinary universities is a clear barrier to ensuring that excellence in IDL is achieved. This is among other barriers such as limited educator interdisciplinary skills, students’ perception of interdisciplinary learning, and lack of infrastructure.^5,6^

### Differentiating alternative learning approaches

To construct effective IDL experiences, it is important to have a clear understanding of the definition of interdisciplinary learning and characteristics of an interdisciplinary learner. There are clear differences between the closely related terminologies of intradisciplinary (or monodisciplinary), cross-disciplinary, multidisciplinary, interdisciplinary, and transdisciplinary (Table 1).^2,10^ Monodisciplinary, as the name suggests, refers to practicing within the boundaries of a discipline. Consequently, monodisciplinary learning prevents students from exploring potential interactions between disciplines and deprives students from the added value gained from exploring a topic from multiple perspectives.^11^ Cross-disciplinary refers to exploring a topic from the view of another discipline while remaining within a discipline boundary. In cross-disciplinary learning, students experience other perspectives; however, they may not actively explore the potential interactions between the disciplines and will remain bound to their original discipline. This learning approach is commonly observed in higher education and in secondary schools. In addition, there is a general misconception that courses that promote cross-curricular learning experience provide their students with IDL experiences and enable them to develop the complex cognitive skills associated with the synthesis of new knowledge. Despite cross-curricular learning using the approach to center the teaching of topics around a common theme, no active encouragement or opportunity is provided within the curriculum to encourage students to explore the links and interconnectedness of the different disciplines and to explore how different disciplines can influence each other to promote a new way of thinking.^8^ In multidisciplinary practice, different disciplines remain within their own boundaries while utilizing the knowledge of different disciplines around a common theme or goal; this practice is commonly seen in healthcare settings where multidisciplinary teams are formed to treat or address a health concern.^1,2,12^ In contrast, in interdisciplinary practice, the links between different disciplines are analyzed and synthesized into a coherent whole to achieve a better understanding of a common theme.^1,2^ Therefore, in IDL, discipline-specific knowledge is integrated around a common theme or problem to achieve a new and more comprehensive perspective of the theme.^3,7,8^ IDL creates a more holistic approach to knowledge acquisition by encouraging the creation of new connections to achieve an enhanced perspective of the topic.^11^ A key characteristic of IDL is the requirement for integration and synthesis of knowledge throughout different stages of learning rather than the process of integration being limited to the end-product. IDL is usually rooted in pedagogical approaches that support holistic and integrative exploration of a theme or a problem and that promote innovative and critical thinking. This showcases that interdisciplinary curriculum design should ideally be anchored in pedagogies that promote deeper reflective thinking around topic areas that are more meaningful and relevant to the students. Such aligned pedagogies may include problem-based learning and debates.^8^ Finally, transdisciplinary practice progresses beyond the consideration of boundaries and synthesis of links between different disciplines around a common theme. Transdisciplinary practice instead seeks to place all disciplines in a constant, evolving integrative system to create a new practice (new discipline) with a humanities and social purpose.^1,2,12^ Recently, in the healthcare setting there has been a call for moving from working in multidisciplinary teams to adopting interdisciplinary or even transdisciplinary practices. This is partially attributed to the recognition of the fact that local and global complex health challenges, which share several features of ‘wicked’ problems, are interlinked and share common features and often have multiple causes and inherent social complexities. Therefore, addressing them requires a transdisciplinary approach, known as One Health, to integrate society and science by including all stakeholders. One health, an integrative approach to health across highly interlinked components, recognizes health professionals as agents for change. It offers a platform to both measure and manage determinants of health that are rarely fully covered by medicine or public health alone.^2,13^

**Table 1.** Disambiguating different disciplinary approaches. Monodisciplinary/intradisciplinary, interdisciplinary, cross-disciplinary, multidisciplinary, interdisciplinary, and transdisciplinary can be easily confused with some terms erroneously used interchangeably.

LEARNING APPROACH	EXPLANATION
Monodisciplinary/intradisciplinary learning	Learning is conducted within the parameters of a single discipline. No exploration of other disciplines or perspectives.
Cross-disciplinary learning	Learning is rooted from one discipline. Unlike intradisciplinary learning, other perspectives are taught (to a limited extent). Contrasted with interdisciplinary learning, this approach grounds understanding from other disciplines within its own needs. For example, Psychology students may learn about sociology theories that are applicable to psychology but not have a wider experience of sociology as a discipline.
Multidisciplinary learning	More than one subject area is taught but they are delivered disparately and are not interlinked. An example would be a British joint degree such as French with Business management.
Interdisciplinary learning	More than one subject area is taught however there is an emphasis on exploring areas of overlap and how the different perspectives can be integrated and complementary towards contributing holistic understanding of topics.
Transdisciplinary learning	While interdisciplinary learning instructs multiple disciplines integratively, transdisciplinary learning attempts to identify new subject areas (eg, human health) that borrow the tools and pedagogical approaches of multiple complementary disciplines.

### Context and background to the curriculum innovation and design

**Figure 1.**
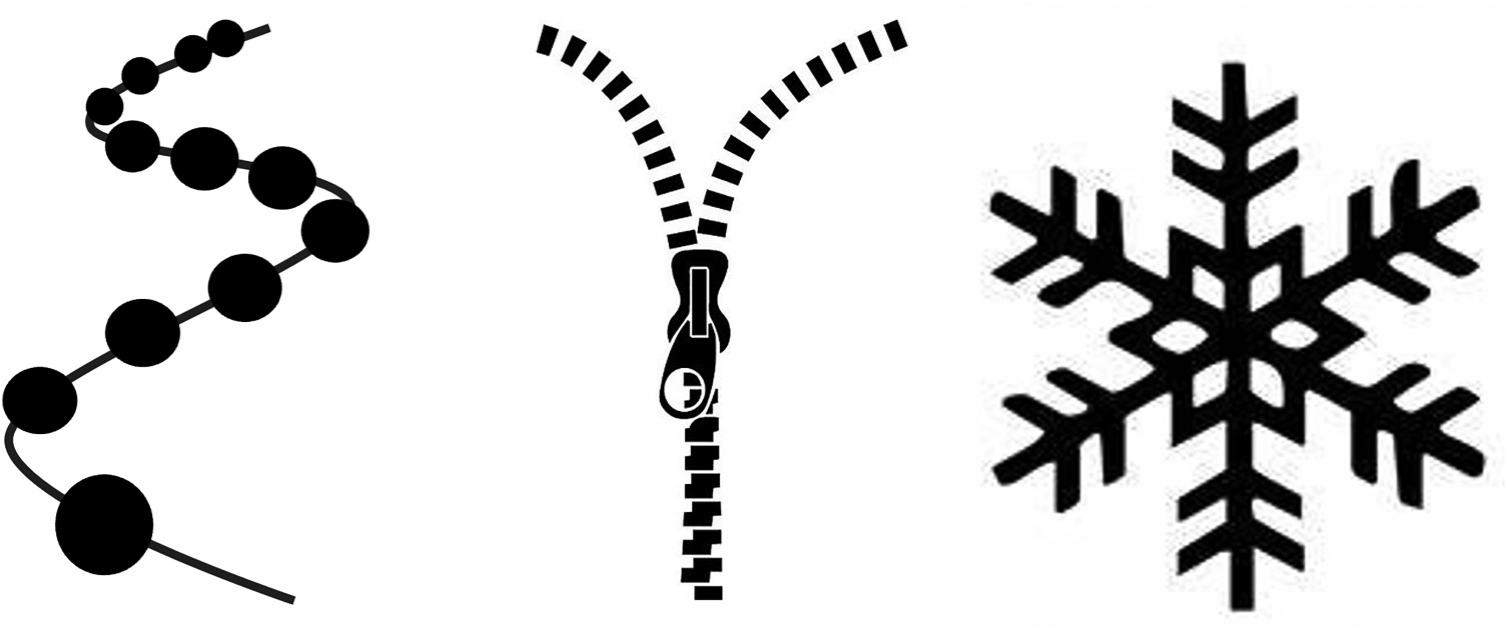
Three models for designing interdisciplinary learning experiences. Lindvig and Ulriksen (2020) proposed 3 models of pearls on a string, the zipper and the snowflake, which underpin the design of IDL with each approach having unique advantages and disadvantages.

**Figure 2.**
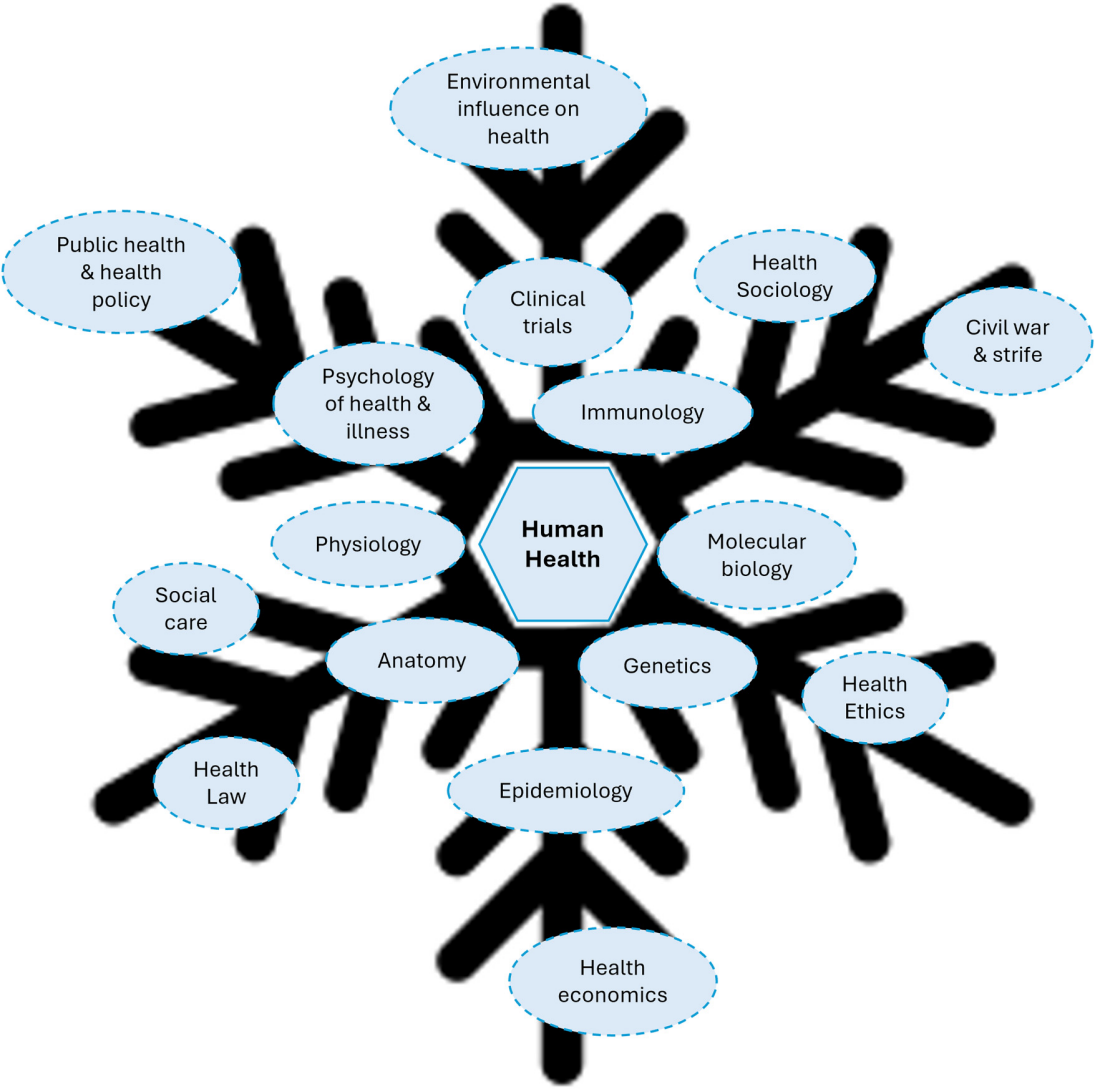
The BSc Health and Medical Sciences discipline areas mapped around the core subject of “human health” to illustrate an example of a snowflake approach. Rather than being a series of topic areas (as in ‘Pearls on a string’), different disciplines can be seen as an interconnected web of perspectives that inform a core area of interest. eg, human health.

Lindvig and Ulriksen^3^ proposed 3 models (Figure 1)—pearls on a string, the zipper and the snowflake— to underpin the design of IDL with each approach having unique advantages and disadvantages. In pearls on a string, the idea is that students will explore individual disciplines separately. However, there is a common theme (string) running through the different disciplines. In the zipper model, similar to the pearls-on-a-string model, students explore different disciplines with the burden of finding a common theme and integrating the different components falling on the student. The snowflake approach, which is usually observed where IDL is delivered using problem solving-based pedagogical approaches, the different discipline-specific elements are organized around a common center (problem or topic area). Figure 2 illustrates how the BSc Health and Medical Sciences adheres to the snowflake approach. Based on the characteristics of IDL, one could argue that learning experiences placing the burden of integration solely on the student, without offering adequate training or space for exploring the integrated nature of encountered disciplines and forming new knowledge, are better classified as cross-curricular learning experiences rather than true instances of IDL.^8^ Therefore, in this study we used a modified snowflake approach where clear “space” and “training” has been superimposed on the snowflake model to promote the practice of integration and cross-linking to design a new interdisciplinary undergraduate course. This approach to designing IDL provides a unique learning experience for students.

BSc Health and Medical Sciences was launched at Warwick Medical School in the academic year 2020–2021 as an interdisciplinary 3-year degree aimed at providing a broad foundation for lifelong learning with a strong emphasis on the development of knowledge, skills, and professional values essential for transdisciplinary practice in applied health promotion for individuals and society. This course has been designed to provide a thorough grounding in health, biomedical and medical sciences concepts, theories, and principles required for supporting an interdisciplinary approach to managing local and global problems in health. The course integrates the 2 broad disciplines of Health Sciences and Medical Sciences to allow a much more comprehensive understanding and exploration of local and global health problems, enabling graduates to adopt a more holistic approach to managing health problems. Medical Sciences involves the study of topics such as molecular biology, genetics and epigenetics, physiology, anatomy, immunology, pharmacology, discovery science, and clinical trials, which aims to promote an in-depth understanding of human biology, health, and disease from the levels of DNA, cells, systems in the human body up to the level of the patient. On the other hand, Health Sciences involves the study of topics such as healthcare systems, ethics, human behaviour, patient safety, psychology of health and illness, sociology, epidemiology, and health economics, which aims to promote an in-depth understanding of health problems encountered from the levels of individuals, societies, and the macrosocial levels of populations and the environment.

**Figure 3.**
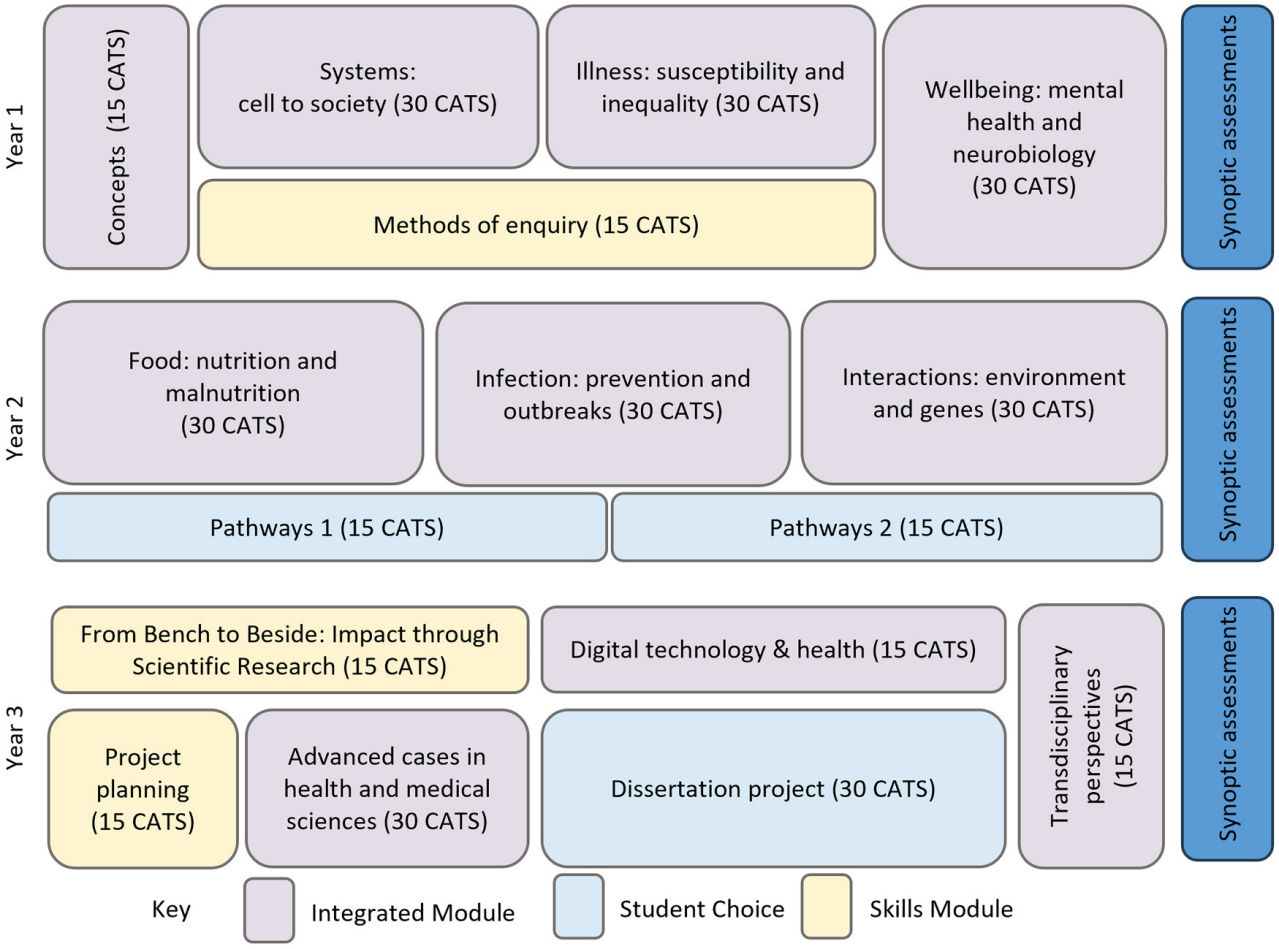
Interdisciplinary course design. All integrated modules are designed and delivered with the intention to explore health problems from multiple perspectives and offer a more holistic approach to managing health problems. This is partly achieved by centralizing module content around three 2-weeklong case studies. As an example, in healthcare systems students explore a patient's case diagnosed with spinal muscular atrophy (SMA). The holistic patient case enables students to integrate various topics from molecular biology, pathology, physiology, anatomy, health ethics, health psychology, health behavior, patient and public involvement (PPI) to healthcare systems. Therefore, in each module students cover various topics that fall under the 2 disciplines of health sciences and medical sciences delivered using various modes of teaching and learning from lectures, group discussions, debates, self-paced interactive online learning material to case-based learning sessions. End-of-year synoptic assessments (or integrative assessments) hold true to the mission of promoting integrated learning and assessment opportunities by allowing students to demonstrate their knowledge and skills in integrating information vertically and horizontally. The Concepts in the Health and Medical sciences module, the first compulsory module undertaken in year 1, has been designed to standardize the background knowledge and understanding of the learners across various disciplines (eg, biology, ethics, health psychology, economics, etc.) covered throughout the course and to familiarize learners with the language, nomenclature, and terminologies of each discipline.

The content in each core module is centered around health problems aligned to the broad areas of physical health, mental health, infectious diseases, noncommunicable diseases, nutrition, and civil strife and displacement. At the time of writing this paper, each integrated module (shown in purple in Figure 3) was coled by experts from each discipline of health sciences and medical sciences with the content of the module also delivered by experts from multiple disciplines aligned to the theme of each module. In addition, during the first 3 years of the course, theme leads aligned to Medical Sciences and Health Sciences ensured a cohesive and balanced representation of both disciplines throughout the modules and course. As a result, in each module health problems are explored from the integrated perspectives of medical and health sciences disciplines using a range of teaching approaches from lectures, group discussions, and concept mapping of module content to debates, seminars, online interactive material, and case-based learning (CBL, Figure 3). As mentioned by Harvie,^8^ problem-based methodologies, such as CBL, debates, and group discussions promote deeper thinking and knowledge acquisition through hypothesizing about solutions to problems, synthesizing new information by exploring connections between various elements, and finally enabling the students to establish their own interdisciplinary purpose. For example, CBL, a signature educational approach for the Warwick Medical School, enables students to apply the knowledge they have acquired in each module to holistic case scenarios and deepen their learning experience.^14^ This also provides students with a safe space to practice interdisciplinary skillsets. Furthermore, CBL provides students with the opportunity to explore multifaceted health problems from the integrated perspectives of health science and medical science. It facilitates students’ exploration of the natural connection between different factors and elements that influence our health. Consequently, a CBL approach promotes vertical and horizontal integration of topics and disciplines covered within and across modules throughout different years of study.^15^

Concepts in the health and medical sciences module, the first compulsory module undertaken in year one, has been designed to standardize the background knowledge and understanding of the learners across various disciplines (eg, biology, ethics, health psychology, economics, etc.) covered throughout the course and to familiarize learners with the language, principles, nomenclature, and terminologies of each discipline. This module is essential as the course is open to students without A-level biology. The content of this module not only provides students with the language, nomenclature, and terminologies of each discipline, it also provides training on essential skills for interdisciplinary learning, academic writing, and general study skills. The module teaches students how to explore the connections between discipline-specific topics and how to synthesize new information, encouraging original thinking. This is partly achieved by hosting a series of skill-based sessions as well as providing students with examples and opportunities to practice integration using case studies. The diverse range of A-level backgrounds (eg, science, technology, engineering, and mathematics (STEM)-related subjects as well as A-levels in languages, art, religious studies, sociology, etc.) of the students further enriches the diversity of the views and perspectives represented in the cohort particularly when it comes to exploring health cases from multiple perspectives during debates or CBL sessions. Students are predominantly British nationals; however, roughly 15%–20% of students are international. All continents have been represented on the course; however, most international students are from Western Europe and China.

**Figure 4.**
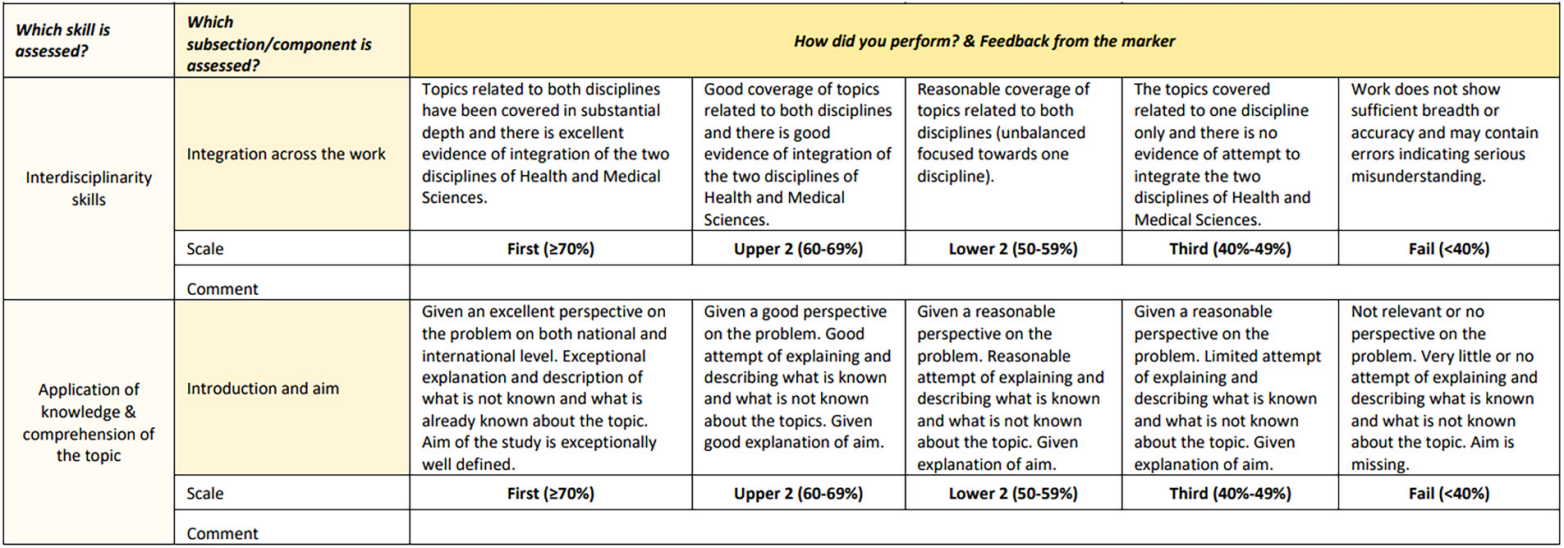
Embedding interdisciplinary practice into assessment criteria. This subsection of a level 4 analytical rubric is an example of a typical rubric used for assignments delivered throughout the course. Assignment tasks require students to practice interdisciplinarity by exploring the topic from multiple perspectives that fall under the broad disciplines of Medical Sciences and Health Sciences. As shown in the figure, a key criterion listed in the rubric is “integration.” The clear descriptors for each standard will guide the student on what is expected from them. Furthermore, the rubric maps each criterion to skills sets, enabling students to assess the type of skills gained and demonstrated with each assessment approach.

In addition to teaching and learning in an interdisciplinary approach, the inclusive assessment strategy of the course uses a diverse array of assessment methods, underpinned by a hybrid model of synopticity as defined by Constantinou.^16^ Integrative assessments (or synoptic assessments) seek to enable students to demonstrate learning against program-level outcomes, showcase their ability to form meaningful connections between different topics and disciplines, and to demonstrate their knowledge and depth of understanding through analysis of information and application of their knowledge.^16^ This ensures that integration, critical thinking, and synthesis of information skills is constantly encouraged and practiced throughout the course to the point that it becomes second nature to the students. Each integrated module is assessed using “local” integrated assessments where assignments and end-of-module exams aim to integrate health sciences and medical science content covered within the module. End-of-year integrated assessments enable learners to demonstrate their ability to explore connections and integrate information horizontally across various modules and course content and to synthesize new information, thereby demonstrating newly acquired insights into a topic. The integrated assessment in year 3, which is delivered as part of the Transdisciplinary Perspective module, enables students to demonstrate their skills in integrating information horizontally and vertically, and to explore health problems through multiple perspectives explored in the 3 years of the course. During the module, students practice progressing beyond the boundaries of disciplines and interdisciplinarity. They are encouraged to adopt a new transdisciplinary approach to managing local and global problems in health rooted in their commitment to benefit communities and society. As demonstrated in Figure 4, the analytical rubrics used throughout the course also promote and value interdisciplinary practice.

### Research aims

The aim of this study is to explore best practices for developing and delivering an interdisciplinary teaching and learning experience from the student perspective. The key focus is to provide a more integrated, holistic experience for the students and to empower them to identify as individuals who can function across the boundaries of various disciplines partly by learning how to practice interdisciplinarity during their studies. The aim of the study was achieved by exploring the views and experience of the inaugural cohort studying the newly established BSc Health and Medical Sciences course at the Warwick Medical School. This course was designed to promote an interdisciplinary learning experience by placing interdisciplinary learning and teaching at the heart of the curriculum design and delivery. The focus group data also allowed exploration of the advantages and pitfalls associated with teaching and learning in a more holistic manner from the student perspective. Overall, the study provides valuable insights and recommendations for educators looking to either design a new interdisciplinary course, introduce interdisciplinary teaching and learning to existing courses or to improve existing interdisciplinary offerings.

## Methods

This small-scale qualitative study was conducted at the Warwick Medical School in February 2022.

### Participants and recruitment

Inclusion criteria mandated participants to be second- or third-year students on the integrated BSc Health and Medical Sciences 3-year degree program. Exclusion criteria entailed first-year students of this degree program or students studying other degrees. It was deemed that first-year BSc HMS students had not experienced enough of the degree to sufficiently convey their experience of the course. Students were contacted via the course resource account and were provided with a study information leaflet. The study information outlined the rationale for the study and what participation in the study involved. Furthermore, the documents outlined procedures for withdrawal from the study, and that responses would be anonymized and decisions regarding participation had no bearing on academic progression. Prior to participation in the study, written consent was obtained from all participants (*n* = 6).

### Data collection

The semistructured focus group interview was conducted according to guidelines and steps outlined by Stalmeijer et al^17^ in a quiet room at the Warwick Medical school with 2 out of the 6 participants joining online using conference audiovisual systems. Focus groups were preferred to individual interviews as they allow symbolic interactionism; an idea that individuals construct shared meanings of phenomena in discussion with others and not in isolation.^18^ Moreover, focus groups allow for more naturalistic discourse compared to interviews due to the social interactions among participants.^19^ To confirm transparency of reporting, an Enhancing the QUAlity and Transparency Of health Research (EQUATOR) Network checklist (the Standards for Reporting Qualitative Research’ checklist) was complied with and is available (Supplementary Table).^20^

A semistructured focus group with 6 participants (all female) was conducted at a time when there was no teaching or assessments to avoid disrupting their studies. Six participants were recruited as this was the number of students who expressed interest and were available to participate. However, the participants represented approximately 15% of the inaugural cohort. The inaugural cohort comprised 29 students with the cohort disproportionally imbalanced toward female students (26 women and 3 men). Four students were British national students, and 2 were international students (of Croatian and Cayman Islands nationalities, respectively). None of these participants had prior experience of IDL. Of the 6 participants, 3 participants were also members of the Student Staff Liaison Committee (SSLC). The SSCL representatives act on behalf of their cohort to ensure the views and voice of their fellow students are captured and represented at the liaison committee meetings with faculty. Inclusion of student representatives among participants meant that participant perspectives were likely to represent those of the cohort more broadly.

The hybrid approach of online and face-to-face discussions was necessitated due to unforeseen, short-notice personal difficulties experienced by 2 participants. Abrams et al^21^ found that virtual focus groups with audiovisual participation (as opposed to text-only) yielded similar data richness to face-to-face focus groups. Hence, it was determined that virtual attendance would not affect data richness. Participants who joined online had their cameras on and could view and hear all participants and facilitators in the room. The focus group was facilitated by LM and with HN asking follow-up questions. Discussions were audio recorded. A semistructured interview guide with open-ended questions was used (Table 2). The interview guide used was not a preexisting (or validated) guide; novel questions were developed to ensure the research aims were met. Due to the small nature of this exploratory study, it was not pilot tested. Probing questions were used where necessary to explore emergent topics or to ask participants for further clarification and information.

**Table 2.** Question areas and questions used for the semi-structured focus group. Question area A: explore and establish students’ descriptions and perceptions of interdisciplinary teaching based on their current knowledge and experience on the course. In addition to where they can identify interdisciplinary teaching in the program at present. Question area B: This theme aims to establish whether, and how, important interdisciplinary teaching is. Questions in the second theme will aim to allow illumination regarding the value and importance students place on interdisciplinary or integrated teaching. Specifically, questions will explore how it may influence their student experience and employability. Question area C: In the final theme, students will be asked questions that allow the opportunity to identify where integrated teaching could be strengthened or introduced in the course as well as requesting examples of additions to the curriculum or pedagogical approach.

Question area A. Student perception of interdisciplinary teaching
Q1. Describe your understanding of what interdisciplinary teaching and learning is? Prompts: What does this phrase mean to you? Can you give any examples? Q2. Which aspects of the course, if any, do you think feature interdisciplinary teaching? Prompts: Where do you see integration of different disciplines? Within module? Across module? Across year? Q3. What approaches would enhance your interdisciplinary thinking and learning? Prompts: Assessment and marking approaches/Delivery of teaching sessions/ Learning opportunities/Skills Q4. Describe or provide examples of teaching approaches that can promote interdisciplinary learning? Prompts: Concept maps/Case-based learning/Interdisciplinary assessment (ie, synoptic exam)/Group discussions
Question area B. Perceived importance of interdisciplinary teaching
Q5. What is your opinion regarding integration/interdisciplinary teaching/learning? Prompts: What impact does it have for you as a student, and your experience of the course? What impact does it have on the course? Q6. What effect does interdisciplinary course have on your employability if any? Prompts: Consider potential acquired skills Q7. Did interdisciplinarity play a role in your degree choice? Prompts: In what way?
Question area C. Improvements for interdisciplinary teaching
Q8. Which of these changes would be most important? Q9. If there was just one thing you could change, what would it be?

### Data analysis

The recorded interviews were transcribed verbatim by student officers recruited for the project. LM checked and confirmed the generated transcript. Data analysis was conducted independently by LM, FN, and HN using reflexive thematic analysis to identify and inform understanding of participants’ perspectives and to structure and report themes within the dataset.^22^ Themes refer to the overarching patterns within the data. To achieve this, the transcript was read by the researchers to enable familiarization. Notable features in the data were then iteratively coded using an inductive approach over 2 rounds of coding for diligence and consistency.^23^ Patterns and connections were actively sought in the codes, and similar codes were amalgamated inductively identifying themes (Supplementary Table 2). The generated codes, themes, and the titles of themes were reviewed and discussed to ensure the final themes accurately represented the data. Researchers maintained reflective notes throughout the analysis period, and these were discussed at researcher meetings. Discrepancies and disagreements between researchers were considered and discussed, enabling consensus to be reached. Once themes and codes were established, the final report was produced.

## Results

The focus group discussion duration was 2 h and 19 min. Considering the small sample, it was unlikely that data saturation was reached. The authors herein align with Varpio et al,^24^ who disputed the use of data saturation as a measure of robustness of qualitative research. Instead, they argue “information power” to be used. This idea contends that data, rather than being saturated, ie, exhaustive (which itself is not objectively discernible), should hold utilitarian value. Considering the quality and richness of the generated data, the sample specificity needing fewer participants,^24^ and gathering insight into a newly established interdisciplinary course, the data were considered sufficient in answering the research questions and providing valuable insight into student experience of IDL and for making subsequent recommendations.

The focus group questions which explored students’ experience, views, and values of learning on an interdisciplinary course, were mapped to 3 broad thematic areas as shown in Table 3. These were “Integration versus co-localisation”; “Learning on an interdisciplinary course”; and “Assessments: A required component of interdisciplinary practice.”

**Table 3.** Overarching question areas. The transcript was coded independently by three individuals. Identified codes were discussed and similar codes were grouped together under an overarching theme. Subthemes were identified within each theme.

THEME	SUBTHEME
Integration versus colocalization	Definition of Interdisciplinarity
	Design: Inherent integration versus special colocalization
Learning on an interdisciplinary course	Pedagogical methods conducive to interdisciplinary learning
	Teaching the language and fundamental principles of each discipline
	Interaction and communication among educators
	Learning environment
	Advantages and challenges
Assessments: A required component of interdisciplinary practice	Function of assessments in an interdisciplinary course
	Assessment method

### Integration versus colocalization

#### Definition of interdisciplinarity

When discussing IDL, Participant 2 defined it as: “*…looking at a single topic from multiple perspectives at the same time to kind of get a holistic idea of what it actually means*.” Participants constructed an understanding of interdisciplinarity as the integration of knowledge from various disciplines around a common theme to achieve novel perspectives and holistic comprehension. Interestingly, this aligns with definitions in the literature.^11^ Furthermore, data also referred to the inseparable relationship between interdisciplinary learning, interdisciplinary teaching, and interdisciplinary practice (assessments) in achieving and promoting interdisciplinarity in education. Notably, Participant 3 opined: “*…Makes me think of the method of teaching, so there's the subject matter and how that all interrelates. But then there's also the different ways of teaching, …*” It appears the experience of IDL extends beyond simply learning content. Discourse highlighted the influence and importance of the modes of learning and teaching in promoting interdisciplinary learning (eg, “case-based learning, debates, and seminars”). Most importantly, the significance of assessment design and tasks in promoting and allowing interdisciplinary practice was also highlighted. Participant 1 emphasizes further: “*…I think like yes, CBL does do it. But in terms of like us, actually putting it into practice, it's probably an assessment.*” Similarly, Participant 2 mentioned it is *“pointless to have the interdisciplinary teaching without interdisciplinary exams, … I wouldn’t have one without the other because I think this is much more effective and kind of long term as well.*” From this, we can infer assessments enable learners to actively apply their interdisciplinary skillsets, providing them with a valuable opportunity and a conducive environment for exploring novel connections and relationships between concepts. What is key in their discourse is that true experience of IDL means they must extend beyond being a receptacle of interdisciplinary content but must also be practitioners of their learning. The notion of practicing being better for “long term” constructs the idea that application of their learning permits a more nuanced understanding of human health and one that is more deeply embedded in their memory. This underlines the idea of a mutually reinforcing and triangular relationship between learning, teaching, and assessments, emphasizing the equal significance of each component in fostering an interdisciplinary practice and mindset in students.

#### Design: Inherent integration versus special colocalization

Participants acknowledged the intrinsic value of engaging with a course designed from conception as an interdisciplinarity learning experience. Furthermore, it was highlighted how experience of learning on an interdisciplinary course can differ from courses where interdisciplinary learning opportunities have been spliced into existing course structures. The difference between joint honors degrees that allow students to undertake modules from a different discipline to a course where the concept of interdisciplinary practice has been embedded into each activity, module, assessment, and student experience was also explored. From the quotes below participants construct the concept of joint honors degrees as being more multidisciplinary rather than true interdisciplinary (“*There isn’t really intentional overlap between their joint degrees,*” *Participant 2)*:

I think it would like completely change like my approach and way of thinking if it was separated. If we had group discussions on the medical sciences I wouldn’t be thinking of like health sciences because it's separate. In my head, it's kind of then more like a joint honors. – P5

I’d be put off taking it if it was a joint honors. And from like friend's experience, I know that like… I talked about history and politics… The departments don’t communicate, for example, and it was all very separate, different assessment types. It's kind of like you’re doing like, half-half to doing each one. I feel like having it both, you’ve got the full focus the whole way through.” – P5

Additionally, it was suggested that in courses where students can enroll onto modules from different departments, the burden of integration largely fell on the learner. The learner is responsible for actively exploring the relationships and interactions between modules from other departments and their degree topic area. The participants also emphasized the importance of consistent and continuous practice of exploring a theme from multiple perspectives throughout the course of their studies as a key component of an authentic interdisciplinary course, with the integrative approach reflected in all activities, including assessments. Participant 2 stated: “*I think real life issues aren’t really separated like that by discipline, it's always, everything's intertwined all the time*,” Ostensibly, part of this need for continual interdisciplinarity within course design comes from the participants’ understanding that interdisciplinarity readies them for real-world problems that they construct as complex and multifaceted necessitating integrative problem solving.

Intriguingly, as exemplified by Participant 2's quote below, it was also highlighted that integration of multiple disciplines within a module by experts provided students with examples of best practices and how this can be achieved. It also showcased the advantage of cross-disciplinary working to achieve a holistic view of a common theme. Clearly participants construct effective IDL as interdisciplinarity being made more apparent to them, and with guidance provided, rather than the responsibility left to them. The mention of academics of “*different backgrounds*” also invokes the notion that part of course design also requires consideration of who will deliver the IDL.

… because we have Health and Medical Sciences in each module, we have lecturers from different backgrounds, and I think that then creates a really multidisciplinary team. And I actually quite like seeing the Health Sciences lead and Medical Sciences lead interacting and how that works and how, even though they have different interests, they can find common ground and find kind of shared overlap over the topics they're researching. – P2

### Learning on an interdisciplinary course

#### Pedagogical methods conducive to interdisciplinary learning

In general, pedagogical methods that allowed the learner the freedom and space to holistically explore the relationship between various concepts and apply the various discipline-specific knowledge they have gained was considered a good approach for promoting interdisciplinary learning. Among the approaches used, CBL, open debates, group discussions, and seminars were listed as most effective for IDL. However, it was noted that the success of each style of teaching heavily depended on the task, structure of the session, and style of delivery. For example, the content of cases used for CBL sessions and style of facilitation (eg, whether allowing students free reign, providing some guidance to avoid students overlooking some learning areas or close management of learning case-based discussions) were listed as important factors in the success of CBL as a pedagogical method for promoting interdisciplinary practice. As mentioned in Participant 6's quote below, cases that were considered unbalanced had greater focus toward one discipline or were heavily facilitated using leading questions were seen as restrictive to holistic practice of interdisciplinarity. A takeaway from this data is that the quality assurance of interdisciplinary teaching is important for it to fulfill its intended objective.

I would say CBL has a big impact when done properly. When done right. So I think for me the main thing has been in some cases it very clearly led you down a medical path and not really lead you down a health path or vice versa. And that's just in terms of what resources are given to you within the case and how the case is written…But when CBL is done right and when there is a mix of Health and Medical Sciences in there, I think that is probably the biggest thing where I can see it and that actually helps. – P6

Below is an interesting observation by Participant 5: When students were provided with excessive structure during seminars, debates, or group discussions, it was regarded as constricting. Use of a heavily structured format in group discussions or debates tended to channel the discussions toward a predefined perspective rather than enabling the learner to comprehensively and freely explore the topic from various viewpoints. Participants construct their understanding of IDL as requiring space to practice independent, creative thinking. Such findings are present in the literature albeit for other assessment types.^25^

I think like on the timetable we have like group discussions and debates. And I’d like imagine and I kind of would assume it was kind of like more like free going…. but because they are quite structured, as soon as we are like given a side of the argument, it's kind of like, it's like less freeing…I feel like we could learn a lot of each other from, like hearing different opinions. – P5

#### Teaching the language and fundamental principles of each discipline

As outlined in Figure 1, the course starts with the Concepts in Health and Medical Sciences module. The discourse below from Participant 4 emerged when discussing this module:

Everyone's got different backgrounds and getting everyone on the same level to be able to get everyone to be comfortable with sharing their opinion was really vital and I didn’t want to do a foundation year so just having that little start – for the people that were very similar like me, they didn’t want a foundation year but they wanted to feel comfortable, it would help on literally all courses I feel. – P4

From this extract, participants expressed the usefulness of ensuring all entry interdisciplinary students learned the essential knowledge for all disciplines within their course in the opening module. This negated any disadvantages some students might have if their previous high school education subjects did not relate to their degree. Studying such a module also precluded the need to study a foundation degree which was perceived as more onerous. As Participant 2 notes in the quotes below, participants also emphasized that this module offered an opportunity to revisit or learn the language, nomenclature, and terminologies specific to each discipline. Another perceived benefit of the module was attributed to inclusion of training in study skills, academic writing, and the gradual acclimatization to the demands of university workload.

I do think that the module is very vital and I think it's kind of necessary to keep it and for me as well like even though I like I did have biology in school, for me it was useful like to learn some words in English like I understood the concepts but then it was really like after that module everything else was familiar to me. When learning new content and like it was good that I didn’t have to go to the dictionary in the third module to understand something. – P2

I think what was very good was that apart from providing introduction into the… both disciplines, it provided introduction into like skills that are useful for the whole university, such as academic writing and plagiarism. – P2

However, as in the quote below, participants did raise a significant point regarding the module's nature as an introduction to various topics and disciplines. They commented on the lack of centralization in teaching around a common theme (ie, a health problem), a key criterion in the definition of interdisciplinary learning, which was evidently present in other integrated modules. This resulted in a less integrated nature for this specific module.

I think concepts is the module where Health and Medical Sciences were the least integrated in teaching, so if that was a bit more integrated… – P2

### Interaction and communication among educators and students

The module co-leadership approach, involving 2 module leads from distinct disciplinary backgrounds for each module was acknowledged as an effective strategy for ensuring that a module is designed and delivered with the intention of fostering interdisciplinary integration and practices. In the extracts below, participants emphasized the most effective IDL approaches involved joint messaging, codelivery of sessions, and contributions from experts across various disciplines within a session. It was noted that having experts from diverse fields present during biweekly discussions, debates, or summary sessions facilitated the exploration of interactions and overlaps across disciplines centered around a common theme. Ostensibly, this observation about teaching deliverers is reminiscent of the findings in the Design: Inherent integration versus special colocalization subsection. Good IDL design requires interdisciplinary collaboration between educators and for this collaborative delivery to be visible to learners.

I find that these group discussions are most helpful when you have more than one person leading the group discussion. Like if you have both the module leads there rather than just one, then they can both sort of pick up on things from their topic areas and the things that the medical science person will know that you’ve covered this in the medical science and the health science person will know that you’ve covered this in the Health Science. And for me those are the ones that have worked the best because it means that we end up talking about more, more of the content, and we end up integrating it more as well. – P3

If you think about, say, A levels, you might be doing topics that might be similar, but I wouldn’t call that interdisciplinary simply because there's no communication between sort of departments or between teachers. And I think that's a key part of it as well. I would say that for it to be classes, interdisciplinary lecturers, slash module leads, whatever, persons in charge would need to communicate with each other, and I think that is probably quite key part. – P6

The style and communication tools used for engaging with the students during their modules were highlighted as an important factor in promoting an interdisciplinary learning environment. For example, module leads who employed concept maps effectively to summarize the content of a week and clarify the interrelationships between weekly sessions aided students in visualizing and reinforcing their understanding of the interconnected nature of subjects that might initially seem disconnected:

Basically I found it really good that every single week they gave us a concept map, but that's just my opinion, I loved the concept map because it like… They basically put every single title of every single lecture. And they said, like, how it all interrelated…. And because on there like it tells you, for example, like how, like reproductive health then affected like someone's risk of cancer, stuff like that. So I would find that helpful. – P1

In general, it was considered best practice to use a variety of pedagogical methods that support interdisciplinary learning. This approach was preferred over relying on a single method to accommodate the diverse needs of learners; however, participants repeatedly construct interdisciplinary teaching to be a collective, intentional effort.

#### Learning environment

The physical learning environment and space greatly influence student experience. Participants mentioned that for interdisciplinary courses it is important that space and timetabling are carefully considered. In the quote below, Participant 1 notes that having sessions related to different discipline areas delivered in a common building implicitly promoted a sense of identity, belonging, and commitment to interdisciplinary practice. It enables better interaction and colearning for students and a sense of togetherness. In courses that encourage interdisciplinary study by permitting students to enroll in modules from other departments, the responsibility for preventing scheduling conflicts and ensuring adequate time between sessions, allowing students to transition from one building to another can be seen as a potential hindrance to fostering a genuine commitment to interdisciplinary learning and teaching. It presents an additional challenge for students to overcome.

… even just the fact that all of our lessons are in one building to me has like an element of interdisciplinarity, because I think like, I’m coming to one like school as it were, like the medical school to do my degree. Whereas like when I think about my friends who have, like, a double honors degree, they are going to like completely different schools. And even then I think that's where it like creates that divide because, they’re having to… I think it was mentioned before, like walk to a different school to go to a particular lecture, whereas here, like it's all here. And I think as well because we all see each other and like outside of lessons, we’re able to have conversations that again like bring everything together… But I think knowing that all of my lessons are in one place is also something that just, like, reminds me that I am on an integrated course. – P1

#### Advantages and challenges

Participants highlighted some of the advantages associated with learning on a new interdisciplinary course. They felt better prepared to consolidate learning by synthesizing ideas from multiple perspectives and better understood the complexity of some of the health challenges and the need for a more holistic experience than compared to cross-disciplinary approaches. Participant 3 stated: “*I think that one of the biggest things is just handling a lot of information and also forming these cross connections in your mind because not all of them are made for you*.” Her discourse suggests the knowledge of multiple disciplines provided them with a more rounded understanding of the complexity of some of the local and global health problems and nurtured their ability to identify more connections and independently integrate disciplines. Her mention of forming cross-connections ties to the intention of IDL endeavoring to develop higher-order cognitive skills, such as analytical and critical thinking. Consequently, they felt better prepared to address the challenges, which they felt set them apart from others.

Learning on a new interdisciplinary course offers the potential for producing graduates capable of transcending discipline-specific boundaries and tackling themes and challenges from a holistic perspective. However, it also presents its own set of challenges. Participants voiced concerns about the future recognition of interdisciplinary skill sets by prospective employers and the appreciation of the value of such a degree (exemplified in the extracts below). The quotes below suggest there is not yet a widespread comprehension (and appreciation) of what interdisciplinary learning entails and imbues in learners; thus, participants perceived it may reduce chances of employability. Additionally, the increased cognitive demand associated with identifying connections between concepts and topics and synthesizing information was occasionally seen as a hurdle.

I actually think that like kind of what our degree is gonna be perceived…. I think it's more about the interdisciplinary bit and what I was saying earlier about someone thinking that we just have superficial knowledge of everything, which then isn’t very useful…. I’m worried that someone is gonna think that I don’t have a deep understanding of anything basically…then, obviously, if it's like a job, it makes more sense to hire someone who has a deep understanding of a specific issue. – P2

My concern would be that they wouldn’t understand the depths of what we’ve learned and that sometimes they might think because it’s Health and Medical Sciences or you’ve just covered like the surface of the biology, which isn’t true, obviously. – P6

### Assessments: A required component of interdisciplinary practice

#### Function of assessments in an interdisciplinary course

Participants recognized assessments as a fundamental component for promotion of interdisciplinary learning and practice. This highlights the importance of carefully considering the assessment modes, tasks, and marking criteria for interdisciplinary courses. In addition, it indicates that students perceive interdisciplinary practice cannot be achieved without considering the function of assessments in the interdisciplinary learning experience.

… by definition, I believe both learning and assessment would have to be there, to be able to call it that. – P4

I find that for me personally, … the best opportunity to really integrate the health and medical science is when I'm doing something like an essay or a presentation or a concept map, because you can really weave them together and you're using this stuff from the lectures, but you're finally sort of merging them and really thinking through the whole story of it. – P3

Participants highlighted the important opportunity assessments provide individual learners to showcase their ability to integrate information and practice and hone their interdisciplinary skills. They felt that at times learning and teaching sessions, such as CBL, emphasized the collective group approach to interdisciplinary practice.

But in terms of assignment because you’re doing that yourself, you have to put everything together. So it's more interdisciplinary teaching and learning in an assignment than it is in CBL because that's usually what we have to do. – P4

### Assessment method

The degree participants study utilizes a variety of assessment methods, of which they felt varied in interdisciplinarity:

but I think that obviously on this course we have lots of different types of assessments and we have different types of exams as well. So with the MCQ exams. We have one exam on one module and we’ll have health and medical science questions, but they’re separate questions. So we have a Health Science question followed by Medical Science question. It's not… I don’t think you really can do it in an MCQ, but those exams are not really interdisciplinary because the questions are very much separate disciplines. But when we do the slightly longer answer questions I think that's probably the chance for us to integrate it more. And obviously the essays and sort of course work style assessments are really the best opportunity. – P3

Not all modes of assessments were highlighted as ideal practice for enabling students to showcase their interdisciplinary skill sets. Overall, assessments that provide learners with greater freedom to synthesize information and explore connections between concepts were deemed more appropriate. Students highlighted that although questions aimed to cover topics from multiple disciplines, multiple-choice questions (MCQs) were least preferable. This also highlights the importance of question writing and drafting stems for questions that integrate topics within a question rather than across questions. However, assignments such as case studies and presentations where the task required them to integrate and synthesize information achieved this aim much better. It was also mentioned that long-answer synoptic or integrative exams provide learners with a better opportunity to explore a topic from multiple perspectives.

## Discussion

The broad aim of this paper was to investigate student perspectives on best practice approaches for the development and delivery of interdisciplinary teaching and learning. Specifically, this study aimed to better understand practice approaches that facilitated student learning that is integrative and empowers their propensity to address multifaceted health problems. Finally, the study also aimed to provide useful insights and recommendations that can be valuable to educators with the objective of developing interdisciplinary learning in new or existing higher education courses.

### Defining IDL and clarifying its utility

Participants of this study firstly clarified their perception of what IDL entailed in the context of Health and Medical sciences; they regarded it as a convergence of multiple disciplines (inclusive of their perspectives, methods, and understanding) applied to complex health problems. This is echoed by the literature which recognizes IDL as an integrative approach whereby learners gain an appreciation of multiple perspectives (and solutions) to various social phenomena.^8,11,26^ Data suggest IDL as an important process to develop higher-order thinking. Indeed, such sentiments are echoed by Ivanitskaya et al^11^ and Lindvig and Ulriksen.^3^ Further, these authors add that IDL enables consideration of various perspectives and epistemologies and allows learners to make higher-order judgments and critical and analytical thinking regarding how and when to invoke knowledge or methods from different disciplines to better address a (health) problem. Lindvig and Ulriksen,^3^ in their review of IDL teaching activities noted most included studies reported using IDL due to the belief monodisciplinary courses insufficiently developed higher education competencies.

Students voiced some concern about presumed employer perception of an IDL course, namely that students would only have superficial knowledge of multiple disciplines and a perceived lack of expertise in each disparate subject area. Clark and Wallace^26^ acknowledged a similar concern for political science students where there was a tacit assumption that students should amass substantive knowledge in the pursuit of increasing their employability. Ivanitskaya et al^11^ rebuked this misperception; they remarked simple knowledge acquisition and recall reflected constrained academic rigor and skill. Consequently, such constraints are insufficient to nurture learners’ ability to translate knowledge into scientific solutions, develop strategic thinking, and ultimately enact tangible change through the use of real-world problem-solving skills. MacLeod and Nagatsu^27^ argued that traditional monodisciplinary education was too rigid to address modern-day environmental, social, and health problems. They further noted that IDL approaches were important for learners to foster higher-order adaptive thinking to meet the demands of complex social problems. Plainly, IDL courses are not intended to develop expertise in any particular discipline but instead aim to train future professionals with high-level thinking skills. Clark and Wallace^26^ emphasized the need to avoid misperceptions or underestimation of IDL’s utility by improving understanding of this intention among learners and educating them about the role broad-based courses play in their metacognitive development.

### Variability in IDL experience

Harvie^8^ acknowledged that although different models of education bear the intent of conferring holistic understanding of complex topics and an accumulation of traditionally discipline-specific epistemologies, not all achieved true IDL. Specifically, Harvie highlighted an intention–implementation gap, whereby many courses were actually offering multidisciplinarity without the prerequisite integration between discipline-specific knowledge and methods to be considered interdisciplinary.^8^ Participants in the present study expressed the perception that they benefitted from greater interdisciplinarity on a course that had interdisciplinarity designed from conception compared to courses such as a joint honors degree. Moreover, the learning environment, where they studied lessons from disciplines within the same departmental building, as opposed to attending lectures from disparate departments in various buildings, helped solidify their sense of the course being integrated. Arguably, that these students lack first-hand experience of a joint honors degree limits their ability to appraise the integrative extent of joint honors courses. However, research lends credence to their perception. Pidgen and Jegede^28^ conducted a qualitative study investigating joint honors students’ perception of their learning experience. They recruited students from 4 UK institutes. The authors found students felt the joint honors structure placed the responsibility of integrating the isolated disciplines on learners; hence, their experience was more akin to a cross-discipline approach^8^ rather than being interdisciplinary. This is particularly true when no effort is made to encourage students to form connections between the discipline-specific knowledge acquired, and when students are not taught the skills required for interdisciplinary or transdisciplinary practice.^28^ Results in the present study found students enjoyed studying a course with integration embedded in its design as it showcased effective areas and examples of integration for them—something Ivanitskaya et al^11^ recommended for IDL. Ivanitskaya et al^11^ further stated that IDL represented more advanced academic skill than merely invoking knowledge from multiple isolated disciplines. Instead, they defined IDL as nurturing critical and analytical thinking, metacognitive skills, and epistemological beliefs.

Clark and Wallace^26^ aptly remarked that prior to integration of disciplinary knowledge, one must first understand how best to organize the knowledge. They further opined that overarching frameworks and theories were important to teach to students as tools for integration and effective IDL. It appears that to foster interdisciplinary thought and skill, students should also have the opportunity to identify and explore the interconnectedness of their studied disciplines as well as guidance in how to do so. Participants in the present study felt their introductory Concepts in Health and Medical science module was crucial in introducing not only the foundation knowledge for the respective disciplines but also the instruction on academic writing skills (inclusive of integration), overarching (socio-ecological) frameworks, and theories guided in implementing integration early.

Participants also noted the use of CBL, the signature pedagogy for the course, as enabling them to consider health problems more holistically and appreciate the multifaceted perspectives required to address them. An example of a case they studied was a COVID-19 case. Students studied different facets of this health problem which invoked different disciplines; spread of virus (epidemiology); nature of the virus (virology); diagnosing COVID-19 symptoms and treatment (clinical and diagnostic medicine); mandates for face masks and lockdowns (public health policy); attitudes/compliance to face mask wear and lockdowns (psychology); vaccine development and distribution (pharmacology and health economics); and inequity of treatment (global health and sociology). The literature has found CBL to fare well for student satisfaction and enjoyment.^14,29,30^ McLean^31^ carried out a review of CBL applications in medical and health-related subjects finding that across 70 studies data suggested CBL was effective in translating theory into practice and consequently improved patient outcomes or healthcare solutions. Hence, this review aligns well with student perception that CBL develops learners beyond simple knowledge acquisition and toward deeper learning. Many studies included in this review paper noted CBL was effective at nurturing critical thinking and addressing health problems and care.

As Lindvig et al^6^ note, implementing IDL in existing courses is more challenging in practice. They observed that traditional Danish undergraduate degrees had rigid structures comprising monodisciplinary modules that do not easily permit incorporation of IDL. It is worth noting many UK courses are similarly structured,^4^ thus achieving interdisciplinarity can be regarded as a more widespread challenge. Another assumption that inhibits provision of IDL experience is that institutional staff are able or willing to instruct in accordance with IDL principles and can also create integrative assessments. Lyall et al^4^ recommended support for teaching staff in various forms: (1) granting access to the literature and good practice research (such as case studies of successful IDL courses or modules); (2) delivering local training, such as tutorials; and (3) allowing enrollment to formal study of IDL, such as postgraduate certificates in higher education (PGCHE) focused on IDL. Participants in the present study emphasized the importance of beginning an interdisciplinary degree with a module designed to standardize the foundational knowledge and understanding of learners, particularly considering the diverse range of A-level subjects undertaken by the cohort. Other interdisciplinary courses may also benefit from such a module as they too are likely to recruit learners with varying educational backgrounds necessitating a need to standardize all entry students.

### Assessment in interdisciplinary learning

Participants acknowledged the key role of assessments in integrated learning. Assessment methods such as essay assignments, design of concept maps, long-answer synoptic or integrative exam questions were highlighted as allowing students to employ integrative thinking and consider health problems more holistically. In particular, essay submissions or long-answer exam questions allowed students the space and scope to explore their own (sometimes novel) connections between studied disciplines. Indeed, Gupta and Motewar^32^ agreed with this view; they noted structured essay questions permitted student creativity in addressing health problems while also assessing analytical thought and reasoning skill. Patil^25^ agreed while also cautioning against “over-structuring” essay questions, as it can reduce innovation in thinking and trivialize the question, reducing the assessment's discriminating ability. Conversely, Patil^25^ also acknowledged that these types of assessment can be less objective when marking and they are time intensive. Razzaq et al^33^ found that coursework provided an opportunity to experience greater IDL especially if the assignments demanded they apply research or knowledge into multifaceted applications or contexts.

Noteworthily, results distinguished that not all assessment methods lend themselves as easily to interdisciplinarity as others. Namely, multiple-choice exam questions tended to be monodisciplinary in the BSc Health and Medical sciences course. It was acknowledged it would require a skilled exam question writer—one who possesses interdisciplinary knowledge to write an appropriate interdisciplinary multiple-choice exam question. The literature suggests the skill needed for item writing for monodisciplinary MCQs is already underestimated with many item writers unable to avoid common pitfalls of nondiscriminating questions, poorly worded stems, and nonfunctioning distractors, as well as not assessing higher-order thinking, such as criticality.^34–36^ Item writers for IDL courses would need to write questions that assess beyond the knowledge/comprehension level and toward higher-order thinking and knowledge application.^36^ Essays and long-answer questions are more open ended than MCQs and thus are easier to invoke IDL thinking from students. The present authors suggest that to write more interdisciplinary MCQs, writing an integrated question stem is most important as this can appropriately guide students to think beyond the confines of singular disciplines. It has already been stated that long-answer questions in synoptic examinations are complementary to the needs of IDL. Due to their open-ended nature, they are easier for question writers to orient students towards consideration of various disciplines and to integrate them in a cohesive answer.^37^ Since participants advocate for the use of appropriate assessments as part of an interdisciplinary approach, synoptic exams represent a suitable, effective assessment. Therefore, they should be considered in the design of IDL courses. Lastly, as it is important that integrative skill is assessed, the Health and Medical Sciences course for most assessments features a rubric criterion assessing this key skill.

### Study limitations

Despite the small sample size of 6, it was worth noting that 3 of the participants were SSLC members; therefore, they were well placed to be cognizant of their peers’ perspectives of the course and to also convey them. The sample had no male participants which may be regarded as a limitation of this study. Çera et al^38^ found gender differences in the perception of the university education quality wherein female students perceived their learning experience more favorably than male counterparts. Consequently, it remains possible that male students on the BSc Health and Medical sciences may not share the same positive attitudes regarding its interdisciplinarity to the same extent. Another drawback that the results here cannot ascertain are whether student demographic variables such as socioeconomic background, prior educational experience of IDL, and linguistic and racial diversity influence their experience of IDL. Furthermore, since the sample comprises data from students studying one specific degree, some recommendations might not be a useful direct fit for other degrees aiming for greater IDL. However, the authors here argue that adaptations are always necessary in translating pedagogical practices to new contexts but the overarching recommendations can still improve IDL value.

Additionally, a hybrid approach comprising of face-to-face discussions with 2 participants participating virtually was necessitated. The literature has found that while online focus groups that are audiovisual (not text-only) produce similar data richness to face-to-face focus groups, potential distractions by virtue of the online medium can cause slight distractions.^21^

Finally, the interview study guide was not pilot tested. A potential benefit missed is that pilot-testing interview guides permits testing the appropriateness of questions and appraising their clarity to participants. However, the researchers were experienced in qualitative methodology and ensured questions were worded openly, nonleading, succinct, and focused (in topic).

### Future research and recommendations for other IDL courses

Future research that can follow from this initial exploratory study can investigate the perspectives of teaching contributors and those involved in curriculum design to discuss their understanding of interdisciplinary learning and to what extent they agree with the findings in this study. Research can also explore whether learner background (socioeconomic background; prior education style and subjects studied; native English speaker vs non-native speaker etc.) influence the experience of, and adaptation to, IDL.

Additionally, it would be useful for similar studies to be conducted for other interdisciplinary courses that are not necessarily health or medical science oriented. IDL courses such as political sciences, environmental sciences, human geography, and urban planning among emerging others can all benefit from the best practice recommendations below. Another research avenue would be to investigate the career destinations or intentions of graduates who have since graduated from the BSc Health and Medical sciences, with a view to also exploring their perceptions of how IDL influenced their graduate occupations (be it further study or employment).

For any course, regardless of subject area(s), that aims to instill a true interdisciplinarity learning experience (as described by McPhee et al^1^) as opposed to being cross-disciplinary, we recommend the following best practice approaches:

- Commencing teaching/Inducting students with an introductory module that instructs the foundational concepts, nomenclature, and epistemologies of the various included disciplines is recommended. Such a module should also aim to equip students with a range of academic skills, eg, academic writing, criticality, integrative thinking, use of frameworks and theories and group work. The module should also aim to introduce students to interdisciplinary learning, including tools for IDL and the perceived benefits of IDL.
- It is important to instruct IDL students how to integrate knowledge as well as to design ample opportunities within modules to practice integration.
- Utilize pedagogical approaches, such as CBL or problem-based learning, to promote IDL opportunities. CBL when used as a lynchpin to draw multiple disciplines can be effective for IDL. For degrees that do not emphasize CBL or problem-based learning yet still require IDL, other effective IDL pedagogical approaches include small group teaching, debates, and seminars.
- Appreciate the role of assessment in students’ approach to learning and implementation of their acquired knowledge. Thus, interdisciplinary assessments that examine the integrative ability of students in application and transdisciplinary thinking (with clear IDL criteria in marking rubrics) is paramount to successful interdisciplinary learning.
- Institutes aiming to incorporate IDL must focus on faculty development in the capacity to deliver IDL as well as design assessments in accordance with IDL. This can be attained through access to best practice approaches, such as IDL skill tutoring or enrollment on interdisciplinarity-focused professional training.

## Conclusion

The data generated from this small-scale qualitative study provide valuable insights into best practices and recommendations for designing and delivering an interdisciplinary course informed by the views and lived experience of students. IDL on the BSc Health and Medical Sciences course is perceived by its students to be an effective IDL experience. Students reported general satisfaction, especially for the breadth of disciplinary perspectives they are exposed to and for the resultant change in their academic skills and thinking. Participants also reported the efficacious use of various pedagogic methods which facilitated integrative learning. One of the aims of this paper was to identify good practice for IDL approaches. This paper functions to report best practice guidance for contemporaries interested in either introducing IDL into current courses or those designing new courses (or modules) that aim to be interdisciplinary.

## Supplemental Material

sj-docx-1-mde-10.1177_23821205241260488 - Supplemental material for Designing an Interdisciplinary Health Course: A Qualitative Study of Undergraduate Students’ Experience of Interdisciplinary Curriculum Design and Learning Experiences Supplemental material, sj-docx-1-mde-10.1177_23821205241260488 for Designing an Interdisciplinary Health Course: A Qualitative Study of Undergraduate Students’ Experience of Interdisciplinary Curriculum Design and Learning Experiences by Leda Mirbahai, Farhan Noordali and Helen Nolan in Journal of Medical Education and Curricular Development
